# A post-conflict vaccination campaign, Central African Republic

**DOI:** 10.2471/BLT.17.204321

**Published:** 2018-06-20

**Authors:** Nicolas Peyraud, Michel Quéré, Geraldine Duc, Corinne Chèvre, Theo Wanteu, Souheil Reache, Thierry Dumont, Robin Nesbitt, Ellen Dahl, Etienne Gignoux, Manuel Albela, Anna Righetti, Marie-Claude Bottineau, Jean-Clément Cabrol, Micaela Sarafini, Samuel Nzalapan, Pauline Lechevalier, Clotilde Rambaud, Monica Rull

**Affiliations:** a Médecins Sans Frontières, 78 rue de Lausanne, Case Postale 1016, 1211 Geneva, Switzerland.; b Health Prefecture, Mambéré-Kadéï, Central African Republic.; c Médecins Sans Frontières, Paris, France.

## Abstract

**Objective:**

To rapidly increase childhood immunization through a preventive, multi-antigen, vaccination campaign in Mambéré-Kadéï prefecture, Central African Republic, where a conflict from 2012 to 2015 reduced vaccination coverage.

**Methods:**

The three-round campaign took place between December 2015 and June 2016 using: (i) oral poliomyelitis vaccine (OPV); (ii) combined diphtheria, tetanus and pertussis (DTP) vaccine, *Haemophilus influenza* type B (Hib) and hepatitis B (DTP–Hib–hepatitis B) vaccine; (iii) pneumococcal conjugate vaccine (PCV); (iv) measles vaccine; and (v) yellow fever vaccine. Administrative data were collected on vaccines administered by age group and vaccination coverage surveys were carried out before and after the campaign.

**Findings:**

Overall, 294 054 vaccine doses were administered. Vaccination coverage for children aged 6 weeks to 59 months increased to over 85% for the first doses of OPV, DTP–Hib–hepatitis B vaccine and PCV and, in children aged 9 weeks to 59 months, to over  70% for the first measles vaccine dose. In children aged 6 weeks to 23 months, coverage of the second doses of OPV, DTP–Hib–hepatitis B vaccine and PCV was over 58% and coverage of the third doses of OPV and DTP–Hib–hepatitis B vaccine was over 20%. Moreover, 61% (5804/9589) of children aged 12 to 23 months had received two PCV doses and 90% (25933/28764) aged 24 to 59 months had received one dose.

**Conclusion:**

A preventive, multi-antigen, vaccination campaign was effective in rapidly increasing immunization coverage in a post-conflict setting. To sustain high coverage, routine immunization must be reinforced.

## Résumé

**Objectif:**

Augmenter rapidement le taux de vaccination des enfants grâce à une campagne de vaccination combinée préventive dans la préfecture de Mambéré-Kadéï, en République centrafricaine, où le conflit qui a sévi de 2012 à 2015 a fait diminuer la couverture vaccinale.

**Méthodes:**

Cette campagne en trois phases s'est déroulée entre décembre 2015 et juin 2016 avec les vaccins suivants: (i) vaccin antipoliomyélitique oral (VPO); (ii) vaccin combiné contre la diphtérie, le tétanos et la coqueluche (DTC), vaccin contre l'*Haemophilus influenza* type B (Hib) et l'hépatite B (DTC–Hib–hépatite B); (iii) vaccin pneumococcique conjugué (VPC); (iv) vaccin contre la rougeole; et (v) vaccin contre la fièvre jaune. Des données administratives ont été recueillies au sujet des vaccins administrés par tranche d'âge, et des enquêtes sur la couverture vaccinale ont été réalisées avant et après la campagne.

**Résultats:**

Au total, 294 054 doses de vaccins ont été administrées. La couverture vaccinale des enfants âgés de 6 semaines à 59 mois a dépassé 85% pour les premières doses de VPO, de vaccin DTC–Hib–hépatite B et de VPC et, chez les enfants âgés de 9 semaines à 59 mois, elle a dépassé 70% pour la première dose du vaccin contre la rougeole. Chez les enfants âgés de 6 semaines à 23 mois, la couverture assurée par la deuxième dose de VPO, de vaccin DTC–Hib–hépatite B et de VPC était supérieure à 58% et celle de la troisième dose de VPO et de vaccin DTC–Hib–hépatite B était supérieure à 20%. Par ailleurs, 61% (5804/9589) des enfants âgés de 12 à 23 mois avaient reçu deux doses de VPC et 90% (25 933/28 764) de ceux âgés de 24 à 59 mois en avaient reçu une dose.

**Conclusion:**

Une campagne de vaccination combinée préventive s'est avérée efficace pour augmenter rapidement la couverture vaccinale dans une région sortant d'un conflit. La vaccination systématique doit être renforcée pour maintenir cette couverture élevée.****

## Resumen

**Objetivo:**

Incrementar rápidamente la inmunización infantil a través de una campaña de vacunación preventiva multiantigénica en la prefectura de Mambéré-Kadéï, República Centroafricana, donde un conflicto entre 2012 y 2015 redujo la cobertura de vacunación.

**Métodos:**

La campaña de tres rondas tuvo lugar entre diciembre de 2015 y junio de 2016 utilizando: (i) vacuna oral contra la poliomielitis (OPV); (ii) vacuna combinada contra la difteria, el tétanos y la tos ferina (DTP), vacuna contra Haemophilus influenza tipo B (Hib) y hepatitis B (DTP-Hib-hepatitis B); (iii) vacuna antineumocócica conjugada (PCV); (iv) vacuna contra el sarampión; y (v) vacuna contra la fiebre amarilla. Se recopilaron datos administrativos sobre las vacunas administradas por grupo de edad y se realizaron encuestas de cobertura de vacunación antes y después de la campaña.

**Resultados:**

En general, se administraron 294.054 dosis de vacuna. La cobertura de vacunación para niños de 6 semanas a 59 meses aumentó a más del 85% para las primeras dosis de OPV, vacuna DTP-Hib-hepatitis B y PCV y, en niños de 9 semanas a 59 meses, a más del 70% para la primera dosis de vacuna contra el sarampión. En niños de 6 semanas a 23 meses, la cobertura de las segundas dosis de OPV, vacuna DTP-Hib-hepatitis B y PCV fue más del 58% y la cobertura de la tercera dosis de vacuna OPV y DTP-Hib-hepatitis B fue superior al 20%. Además, el 61% (5804/9589) de los niños de 12 a 23 meses habían recibido dos dosis de PCV y el 90% (25933/28764) de entre 24 y 59 meses habían recibido una dosis.

**Conclusión:**

Una campaña de vacunación preventiva, con múltiples antígenos, fue efectiva en el rápido aumento de la cobertura de inmunización en un contexto posterior al conflicto. Para mantener una cobertura alta, se debe reforzar la inmunización rutinaria.

## ملخص

**الهدف:**

زيادة تمنيع الأطفال بسرعة من خلال حملة تطعيم وقائية متعددة المستضدات في مقاطعة مامبيري كاديي، بجمهورية أفريقيا الوسطى، حيث أدى الصراع الذي دار من عام 2012 إلى عام 2015 إلى الحد من تغطية التطعيمات.

**الطريقة:**

تم إطلاق حملة تتكون من ثلاث جولات ما بين كانون الأول/ديسمبر 2015 وحزيران/يونيو 2016 باستخدام: (1) لقاح شلل الأطفال الفموي (OPV)؛ و(2) اللقاح المختلط بالدفتيريا والتيتانوس والسعال الديكي (DTP)، ولقاح *المستدمية النزلية * من النوع ب (Hib)، والتهاب الكبد (ب) (DTP-Hib-التهاب الكبد ب)؛ و(3) لقاح المكورات الرئوية المقترن (PCV)؛ و(4) لقاح الحصبة؛ و(5) لقاح الحمى الصفراء. تم جمع البيانات الإدارية عن اللقاحات التي وفقاً للمجموعة العمرية، كما تم إجراء مسوح تغطية التطعيم قبل وبعد الحملة.

**النتائج:**

بشكل إجمالي، ﺗﻢ تقديم 294054 ﺟﺮﻋﺔ ﻣﻦ اﻟﻠﻘﺎح. زادت تغطية التطعيم للأطفال الذين تتراوح أعمارهم بين 6 أسابيع و59 شهرًا إلى أكثر من 85٪ للجرعات الأولى من لقاحOPV ، ولقاح DTP-Hib-التهاب الكبد ب، وPVC، وفي الأطفال الذين تتراوح أعمارهم بين 9 أسابيع إلى 59 شهرًا، زادت إلى أكثر من 70٪ للجرعة الأولى من الحصبة. في الأطفال الذين تتراوح أعمارهم بين 6 أسابيع و23 شهرًا، كانت تغطية الجرعات الثانية من لقاح OPV، ولقاح DTP-Hib-التهاب الكبد ب، وPCV أكثر من 58٪، وكانت تغطية الجرعات الثالثة من لقاح OPV، ولقاح DTP-Hib-التهاب الكبد ب، أكثر من 20٪. وعلاوة على ذلك، تلقى 61٪ (5804/9589) من الأطفال الذين تتراوح أعمارهم بين 12 و23 شهرًا، جرعتين من PCV، كما تلقى 90٪ (25933/28764) من الأطفال الذين تتراوح أعمارهم بين 24 إلى 59 شهرًا، جرعة واحدة.

**الاستنتاج:**

كانت حملة التطعيم الوقائية متعددة المستضدات فعالة في زيادة تغطية التمنيع بسرعة في فترة ما بعد الصراع. للحفاظ على تغطية عالية، يجب تعزيز التمنيع الروتيني. ****

## 摘要

**目的:**

通过在中非共和国曼贝雷-卡代省实行一次预防性的多抗原疫苗接种运动，以快速提高儿童疫苗接种量。曼贝雷-卡代省曾于 2012 年至 2015 年间由于冲突而减少了疫苗接种覆盖率。

**方法:**

2015 年 12 月至 2016 年 6 月举行了三轮疫苗接种运动，分别为：(i) 口服脊灰减毒活疫苗 (OPV)；(ii) 百白破疫苗 (DTP) 、乙型流感嗜血杆菌疫苗 （Hib） 和乙型肝炎疫苗 (DTP–Hib–hepatitis B) 的混合疫苗；(iii) 肺炎球菌结合疫苗 (PCV)；(iv) 麻疹疫苗；(v) 黄热病疫苗。搜集根据年龄段管理的疫苗相关管理性数据，并在接种运动前后对疫苗接种覆盖率进行了调查。

**结果:**

共计接种 294,054 剂疫苗。首次接种口服脊灰减毒活疫苗 (OPV)、百白破、乙型流感嗜血杆菌和乙型肝炎 (DTP–Hib–hepatitis B) 混合疫苗和肺炎球菌结合疫苗 (PCV) 的 6 周至 59 个月儿童的疫苗接种覆盖率增长至 85% 以上。首次接种麻疹疫苗的 9 周至 59 个月儿童的疫苗接种覆盖率增长至 70% 以上。二次接种口服脊灰减毒活疫苗 (OPV)、百白破、乙型流感嗜血杆菌和乙型肝炎 (DTP–Hib–hepatitis B) 混合疫苗和肺炎球菌结合疫苗 (PCV) 的 6 周至 23 个月儿童的疫苗接种覆盖率为 58% 以上。三次接种的覆盖率为 20% 以上。另外，12 至 23 个月儿童中有 61% (5804/9589) 接种过两次肺炎球菌结合疫苗 (PCV) ，24 至 59 个月儿童中有 90% 接种过一次此疫苗。

**结论:**

预防性的多抗原疫苗接种运动能够快速有效地提高冲突后的疫苗接种覆盖率。为了保持高覆盖率，必须加强常规免疫接种。****

## Резюме

**Цель:**

Быстрый рост уровня иммунизации детей посредством превентивной кампании с использованием многоантигенной вакцины в префектуре Мамбере-Кадеи, Центрально-Африканская Республика, где конфликт, длившийся в период с 2012 по 2015 год, сократил охват вакцинацией.

**Методы:**

В период с декабря 2015 года по июнь 2016 года проводилась трехэтапная кампания, в рамках которой использовались следующие вакцины: (i) оральная полиомиелитная вакцина (ОПВ); (ii) комбинация вакцины против дифтерии, столбняка и коклюша (АКДС) и вакцины против гепатита В и гемофильной палочки типа В (*Haemophilus influenza* type B, Hib) (АКДС-Hib-гепатит B); (iii) пневмококковая конъюгированная вакцина (ПКВ); (iv) противокоревая вакцина; (v) вакцина против желтой лихорадки. Были собраны административные данные о введенных вакцинах в разных возрастных группах и обследованиях охвата вакцинацией, которые проводились до и после кампании.

**Результаты:**

В целом было введено 294 054 дозы вакцин. Охват вакцинацией для детей в возрасте от 6 недель до 59 месяцев увеличился до более 85% для первых доз ОПВ, АКДС-Hib-гепатит B и ПКВ, а у детей в возрасте от 9 недель до 59 месяцев — до более 70% для первой дозы противокоревой вакцины. У детей в возрасте от 6 недель до 23 месяцев охват второй дозой ОПВ, АКДС-Hib-гепатит B и ПКВ составил более 58%, а охват третьей дозой ОПВ и АКДС-Hib-гепатит B составил более 20%. Кроме того, 61% детей (5804/9589) в возрасте от 12 до 23 месяцев получили две дозы ПКВ, и 90% детей (25933/28764) в возрасте от 24 до 59 месяцев получили одну дозу.

**Вывод:**

Профилактическая кампания с использованием многоантигенной вакцины была эффективной для быстро растущего охвата иммунизацией в постконфликтных ситуациях. Для поддержания высокого уровня охвата необходимо укрепить плановую иммунизацию.

## Introduction

The conflict in the Central African Republic between the end of 2012 and 2015 had a negative effect on the health of the population. By 2015, the mortality rate in children younger than 5 years was 130 per 1000 live births, one of the highest in the world.^1^ The situation developed into both an acute and a chronic humanitarian emergency, which severely disrupted the health system and limited access to medical care and vaccination programmes.^1^^,^^2^ By 2015, the overall security situation in the country had improved.^3^^,^^4^

Before the conflict, the logistic, financial and human resources available for the Expanded Programme on Immunization (EPI) in the country were already insufficient, with only 395 (48%) of the 815 peripheral health centres providing immunization for the population.^5^ During the armed conflict, parts of the vaccine cold chain and vehicles were plundered and about a quarter of health facilities were destroyed, which further aggravated a precarious situation.^5^ The consequent immediate decline in vaccination coverage resulted in the measles epidemics.^1^^,^^5^^,^^6^ Moreover, as the epidemiological surveillance system was also weakened, it is highly likely that diseases targeted by the EPI were under-reported.

In 2015 and 2016, vaccination activities by the country’s health ministry were conducted according to the EPI and targeted only children younger than 1 year: (i) at birth, bacille Calmette–Guérin (BCG) vaccine and oral poliomyelitis vaccine (OPV) were given; and (ii) at 6, 10 and 14 weeks of age, infants received three doses of a combination of vaccines against diphtheria, tetanus and pertussis (DTP), *Haemophilus influenzae* type B (Hib) and hepatitis B (DTP–Hib–hepatitis B) as well as OPV and pneumococcal conjugate vaccine (PCV). In addition, one dose each of measles vaccine and yellow fever vaccine were given between 9 and 12 months of age. The country’s comprehensive multiyear vaccination plan for 2011 to 2015 included periodic supplementary immunization activities against poliomyelitis, measles, yellow fever, neonatal tetanus and meningitis.^5^ Despite a measles vaccination catch-up campaign in children aged 6 months to 15 years in 2005 and 2006 and follow-up campaigns in children aged 6 months to 5 years, which were planned for every 2 years, reactive vaccination campaigns were also necessary in response to outbreaks, as occurred in May 2014 in Mambéré-Kadéï prefecture.^5^ At the national level, vaccination coverage in 2011 and 2012, before the conflict, was: (i) 47% for the third OPV dose; (ii) 69% for the first DTP–Hib–hepatitis B vaccine dose and 47% for the third dose; (iii) 49% for the first measles vaccine dose; (iv) 48% for yellow fever vaccine; and (v) 74% for BCG vaccine. In the first year of the conflict (2013), coverage dropped dramatically to: (i) 23%; (ii) 35% and 23%; (iii) 25%; (iv) 24%; and (v) 37%, respectively.^7^

In Mambéré-Kadéï prefecture, where Médecins Sans Frontières was working, the internally displaced population was estimated at 22 200 in May 2015.^8^ Although the security situation had become less tense after the majority of the Muslim population fled to Cameroon, it remained fragile.^3^^,^^4^ In 2014, the prevalence of severe acute malnutrition was over 2%.^9^ Before the conflict, in 2012, Mambéré-Kadéï ranked sixth lowest among all 16 prefectures for the third dose of DTP–Hib–hepatitis B vaccine. Vaccination coverage that year was: (i) 46% for the third OPV dose; (ii) 59% for the first DTP–Hib–hepatitis B vaccine dose and 47% for the third; (iii) 59% for the first measles vaccine dose; (iv) 60% for yellow fever vaccine; and (v) 59% for BCG vaccine (Dumolard LB, World Health Organization [WHO], personal communication, 2018). Coverage dropped during the conflict and, by 2015, was: (i) 37%; (ii) 58% and 38%; (iii) 47%; (iv) 42%; and (v) 46%, respectively (Dumolard LB, WHO, personal communication, 2018). Children who were younger than 1 year between 2013 and 2015 during the conflict and who were not vaccinated during this period did not have the opportunity to catch up on vaccinations afterwards because, in the Central African Republic, the EPI was limited to children younger than 1 year.^5^ There was, therefore, a cohort of children aged 2 to 3 years in the prefecture who had completely missed the opportunity to be vaccinated.^5^

In 2015 and 2016, Médecins Sans Frontières worked with the health ministry to quickly improve immunization coverage in Mambéré-Kadéï: a preventive multi-antigen mass vaccination campaign that targeted all eligible children younger than 5 years was organized. Here we report on the feasibility and operational details of the campaign.

## Methods

The preventive, multi-antigen, mass vaccination campaign targeted 323 towns and villages in five of the seven subprefectures of Mambéré-Kadéï: Amada Gaza, Berbérati, Dédé Mokouba, Gamboula and Sosso-Nakombo. The total area of the five subprefectures was 16 991 km^2^ and their total estimated population in 2015 was 274 332, giving a density of 16 inhabitants/km^2^.^10^ Another Médecins Sans Frontières unit was operating in the two remaining subprefectures. In the five campaign subprefectures, the number of children aged 6 weeks to 5 years targeted for vaccination was estimated at 45 647 from census data.

### Vaccination strategy

We opted to conduct a preventive, mass vaccination campaign because very low vaccination coverage had to be increased rapidly and several birth cohorts of children younger than 5 years had completely missed the EPI. In addition, this strategy was in line with recommendations of WHO’s framework for vaccination in acute humanitarian emergencies.^11^ An alternative approach focusing solely on improving the EPI would not have been sufficient because, in the Central African Republic, it would have covered only children younger than 1 year.^5^^,^^6^

After several meetings between Médecins Sans Frontières and EPI managers at national and district levels, an overall strategy was proposed by Médecins Sans Frontières and approved by the health ministry and EPI managers. Médecins Sans Frontières took responsibility for the campaign’s logistics and financial costs. Vaccines for children younger than 12 months were partially provided by the health ministry, PCV was donated by industry and the remainder were provided by Médecins Sans Frontières. Vaccination teams were composed of Médecins Sans Frontières and health ministry staff.

**Fig. 1:**
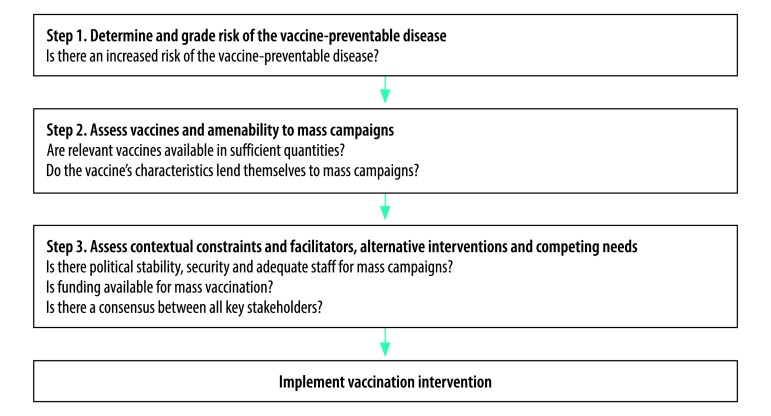
Decision-making steps on vaccine use in acute humanitarian emergencies

Note: The steps are based on the World Health Organization’s framework for vaccination in acute humanitarian emergencies.^11^

We applied WHO’s decision-making framework for vaccination in acute humanitarian emergencies to select the final five vaccines for the campaign (Fig. 1):^11^ OPV, combination DTP–Hib–hepatitis B vaccine, 13-valent PCV, measles vaccine and yellow fever vaccine. This selection corresponded to all EPI antigens in the national immunization schedule except for BCG. MenAfriVac and rotavirus vaccines were not included in the EPI calendar at that time and could not be provided. Moreover, inactivated polio vaccine could not be included, because national and international stocks were limited and its introduction into the EPI had been delayed. The campaign followed EPI’s recommended vaccination schedule for infants, but also included catch-up vaccination with the same antigens for children aged 12 to 59 months, in accordance with WHO’s recommendations.^11^

The first round of the campaign took place between 18 December 2015 and 31 January 2016. Three vaccines were offered to children aged 6 weeks to 59 months: OPV, DTP–Hib–hepatitis B and PCV. In the second round, which took place between 23 February and 4 April 2016, measles vaccine was added for children aged 9 to 59 months. In the third round, between 24 April and 6 June 2016, yellow fever vaccine was added but restricted to children aged 9 to 11 months, because of national and worldwide shortages. In addition, vaccination teams distributed vitamin A in the first round, conducted nutritional screening in the second round and provided soap in the second and third rounds. Caregivers also received paracetamol for use with side-effects, such as fever and pain at the injection site. An interval of 8 weeks was maintained between each of the three rounds.

The campaign was selective, it included all children without a record of immunization on their vaccination card, regardless of their caretaker’s recall. As EPI schedules are complex, with children receiving 14 different vaccines during five or more visits, relying on a caretaker’s recall of a child's vaccination history may have resulted in missed opportunities.^12^^,^^13^ The locations of the vaccination sites were selected on the basis of population density and geographical distribution.

### Data collection and analysis

The principal outcome was vaccination coverage of children aged 6 weeks to 59 months. Administrative data were collected during the campaign as the number of vaccines administered by age group, data on routine EPI activities running in parallel were not included. Data were collected on tally sheets and android applications and analysed using Excel 2003 (Microsoft Corporation, Redmond, United States of America). In addition, two vaccination coverage surveys were carried out in the five subprefectures included in the vaccination campaign: the first took place before the campaign, in December 2015, and the second, after the campaign, in June and July 2016. Gender and wealth disparities were not assessed. The surveys used a two-stage, cluster random sample method, as outlined in WHO’s *Vaccination Coverage Cluster Surveys Reference Manual*.^14^ In the first stage, clusters (i.e. villages) were selected with a probability proportional to size; in the second stage, households in each village were randomly selected according to the modified WHO–EPI method.^14^ Data on each child’s vaccination history were based on the caretaker’s recall and the vaccination card, if available. Reasons for nonvaccination were also noted. For the baseline survey, data were transferred from paper to Excel 2003 and analysed in Epi Info (Centers for Disease Control and Prevention, Atlanta, USA). Data from the postcampaign survey were collected electronically and analysed using Stata v. 12.0 (StataCorp LP., College Station, USA). Data from the caretaker’s recall and the vaccination cards took sampling weights into account and were reported with 95% confidence intervals (CIs). Both surveys were conducted with the permission and cooperation of the local public health ministry. In addition, postcampaign vaccination coverage was also estimated separately by adding precampaign survey results to coverage derived from administrative data.

## Results

On average, 176 vaccination sites were involved in each vaccination round. The number of sites was increased slightly in each round to improve accessibility because of low attendance at locations that were too far from children’s homes. Each round took 6 weeks to complete. There were no major delays in implementation and no stock ruptures or major adverse events were recorded. The campaign employed 316 staff and administered 294 054 vaccine doses (Table 1) in 98 729 consultations. Average attendance during the three rounds was 72% and 97% of children who presented at a vaccination site received at least one vaccine. Of the 98 729 children attending the campaign, 16 361 (17%) were younger than 1 year. A mean of nine vaccine doses were administered per child during the three rounds.

**Table 1:** Vaccinations by age group, preventive mass vaccination campaign, Mambéré-Kadéï prefecture, Central African Republic, 2015–2016

Vaccine	No. vaccinations administered			
	Age group			
	6 weeks to 11 months	12 to 23 months	24 to 59 months	6 weeks to 59 months
OPV	13 823	16 920	62 594	93 337
Combined DTP–Hib–hepatitis B vaccine	14 034	26 257	44 155	84 446
PCV	14 107	15 934	30 744	60 785
Measles vaccine	2 625	9 520	42 848	54 993
Yellow fever vaccine	408	57	28	493
**Total**	**44 997**	**68 688**	**180 369**	**294 054**

DTP: diphtheria, tetanus and pertussis; Hib: *Haemophilus influenzae* type B; OPV: oral poliomyelitis vaccine; PCV: pneumococcal conjugate vaccine.

Administrative data collected during the campaign showed that immunization coverage was greater than 85% for the first doses of OPV, DTP–Hib–hepatitis B vaccine and PCV in children aged 6 weeks to 59 months and greater than 70% for the first dose of measles vaccine in children aged 9 weeks to 59 months. In children aged 6 weeks to 23 months, coverage of the second doses of OPV, DTP–Hib–hepatitis B vaccine and PCV increased to over 58% and coverage of the third doses of OPV and DTP–Hib–hepatitis B vaccine increased to over 20% (Table 2). In addition, 61% (5804/9589) of children aged 12 to 23 months received two doses of PCV and 90% (25933/28764) of those aged 24 to 59 months received one dose, which represented full vaccination of children in these age groups.

**Table 2:** Vaccination coverage, preventive mass vaccination campaign, Mambéré-Kadéï prefecture, Central African Republic, 2015–2016

Vaccine and dose	Vaccination coverage										
	Administrative data collected during the campaign, no, (%)^a^				Surveys before and after the campaign^b^						Estimated coverage for children aged 12 to 23 months after the campaign, %^c^
	Children aged 6 weeks to 11 months *n* = 7 295	Children aged 12 to 23 months *n* = 9 589	Children aged 24 to 59 months *n* = 28 764		Before the campaign in children aged 12 to 23 months *n* = 291		After the campaign in children aged 12 to 23 months *n* = 283		Change in coverage from before to after the campaign		
					No.	% (95% CI)	No.	% (95% CI)	Percentage points (95% CI)		
**OPV**											
Dose 1	8 065 (110.6)	8 271 (86.3)	30 971 (107.7)		50	17.2 (10.6 to 24.4)	219	77.4 (69.2 to 83.9)	60.2 (48.7 to 71.77)		103.8
Dose 2	4 236 (58.1)	5 702 (59.5)	21 915 (76.2)		44	15.1 (9.3 to 21.6)	170	59.9 (52.5 to 66.9)^d^	44.7 (32.7 to 56.8)		74.9
Dose 3	1 522 (20.9)	2 947 (30.7)	9 708 (33.8)		41	14.1 (8.4 to 20.4)	104	36.7 (30.2 to 43.8)	22.7 (10.6 to 34.7)		45.1
**Combined DTP–Hib–hepatitis B vaccine**											
Dose 1	8 185 (112.2)	8 392 (87.5)	34 650 (120.5)		49	16.8 (10.3 to 23.6)	217	76.7 (68.4 to 83.3)	59.8 (10.6 to 34.7)		104.5
Dose 2	4 306 (59.0)	5 745 (59.9)	8 658 (30.1)		42	14.4 (8.5 to 20.6)	169	59.5 (52.0 to 66.6)^d^	45.1 (33.3 to 57.1)		74.4
Dose 3	1 543 (21.2)	2 183 (22.8)	823 (2.9)		37	12.7 (7.0 to 18.6)	71	25.1 (19.2 to 32.1)	12.4 (0.5 to 24.2)		35.6
**PCV**											
Dose 1	8 116 (111.3)	8 304 (86.6)	25 933 (90.2)		48	16.5 (10.4 to 22.8)	216	76.3 (68.3 to 82.8)	59.8 (48.2 to 71.4)		103.2
Dose 2	4 279 (58.7)	5 804 (60.5)	4 027 (14.0)^e^		43	14.8 (8.8 to 20.9)	159	56.0 (48.5 to 63.2)^d^	41.2 (29.1 to 53.3)		75.4
Dose 3	1 712 (23.5)	1 826 (19.0)^e^	784 (2.7)^e^		41	14.1 (8.4 to 20.0)	66	23.3 (17.6 to 30.2)^e^	9.2 (−2.5 to 21.0)^e^		33.2^e^
**Measles vaccine**											
Dose 1	2 625 (125.9)^f^	6 829 (71.2)	26 836 (93.3)		75	25.8 (16.9 to 35.2)	201	70.8 (64.3 to 76.6)^d^	45.0 (33.8 to 56.2)		97.2
Dose 2	0 (0.0)^e,f^	2 691 (28.0)	16 012 (55.7)		50	17.2 (8.9 to 25.9)	99	35.0 (29.1 to 41.4)	17.8 (6.0 to 29.6)		59.5
**Yellow fever vaccine**	408 (19.6)^f^	57 (0.6)^e^	28 (0.1)^e^		33	11.3 (6.7 to 16.8)	23	8.4 (4.8 to 14.2)^e,g^	−3.0 (−14.3 to 8.3)^e^		12.4^e^

CI: confidence interval; DTP: diphtheria, tetanus and pertussis; Hib: *Haemophilus influenzae* type B; OPV: oral poliomyelitis vaccine; PCV: pneumococcal conjugate vaccine.

^a^ For administrative data, estimated coverage could be more than 100% in several situations. For example, vaccination doses may not have been correctly attributed as first, second or third doses because previously vaccinated children may have returned without vaccination cards, with the result that their second or third doses were regarded as first doses.

^b^ Vaccination history in the surveys was based on the caretaker’s recall and the vaccination card, if available.

^c^ From survey and administrative data. Vaccination coverage after the campaign was estimated by adding precampaign survey results to coverage derived from administrative data.

^d^ The denominator was 284.

^e^ These data are not representative because vaccination was not indicated for all children in the age group.

^f^ The denominator was 2085.

^g^ The denominator was 275.

Note: Inconsistencies arise in some values due to rounding.

**Fig. 2:**
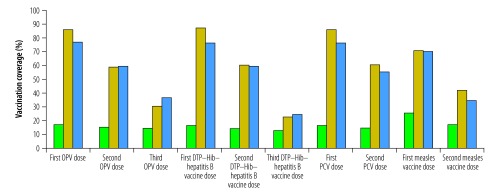
Vaccination coverage of children aged 12 to 23 months before and after preventive mass vaccination campaign, Mambéré-Kadéï prefecture, Central African Republic, 2015–2016

DTP: diphtheria, tetanus and pertussis; Hib: *Haemophilus influenzae* type B; OPV: oral poliomyelitis vaccine; PCV: pneumococcal conjugate vaccine.

Notes: Vaccination history in the surveys was based on the caretaker’s recall and the vaccination card, if available. Administrative data collected during the vaccination campaign did not include data on parallel activities under the Expanded Programme on Immunization.

Table 2 and Fig. 2 show that the postcampaign vaccination coverage survey yielded similar results to the administrative data. According to survey data, the campaign increased coverage for all antigens in targeted children except for yellow fever vaccine (Table 2). Moreover, the postcampaign survey found that 56% (159/284) of children aged 12 to 23 months had received complete PCV vaccination, as had 76% (272/357) of those aged 24 to 59 months. In that survey, the main reasons given for children’s nonattendance for all vaccinations except yellow fever vaccination were: (i) “child or household absent during vaccination” (mean: 37 to 46%; 95% CI: 31 to 53) and (ii) “child's caretaker did not have time” (mean: 22 to 24%; 95% CI: 15 to 34).

## Discussion

In a chronic humanitarian emergency in the Central African Republic, our preventive, multi-antigen, vaccination campaign resulted in a substantial and rapid increase in vaccine coverage after three vaccination rounds, thereby potentially decreasing the burden of targeted diseases and the risk of epidemics.^11^^,^^15^^,^^16^ In addition, most of the beneficiaries were aged 12 to 59 months and thus benefited from catch-up vaccination that would have otherwise been unavailable to them.

It is possible that actual vaccination coverage might have been higher than we found from administrative data. Some doses recorded as first doses were probably, in reality, second or third doses because children presenting without a vaccination card may have been previously immunized. Hence, coverage of the first dose may have been lower than that indicated by administrative data and coverage of second or third doses may have been higher. The absence of an improvement in yellow fever vaccine coverage occurred, because vaccination was indicated only for children aged 9 to 11 months. 

The study had several limitations. The vaccination coverage surveys were based on vaccination cards and caretaker recall, which may not have been reliable. A 2013 systematic review found that coverage estimates based on vaccination cards tended to be too low (with a high positive and a low negative predictive value), whereas estimates based on recall tended to be too high, coverage based on the combination could be either overestimated or underestimated.^17^ Possession of a vaccination card has been associated with higher coverage, possibly because it reflects greater commitment, or access, to vaccination. However, a substantial proportion of the population does not keep cards.^12^^,^^13^^,^^17^ Moreover, replacement cards provided for lost cards may omit recall data, thereby contributing to underestimates of vaccination coverage. In addition, the interpretation of survey results depends on the timing of vaccination relative to the child’s age: children older than 12 months require fewer doses than those younger than 12 months (for instance, the second and third PCV doses are not indicated for children older than 23 months). We were unable to take this into account in our surveys. Caution is also required when comparing our coverage estimates with those of other surveys, because of differences in methods.^18^ Another limitation is that the denominator we chose for calculating coverage and attendance (i.e. the population in last census in 2003) may have been inaccurate, because of population movements during and after the armed conflict.^2^^,^^5^ However, we do not believe that possible population movements would have had a substantial effect on our estimates, given the location and timing of our vaccination campaign.

Achieving high vaccination coverage for vaccines requiring three doses (i.e. OPV, DTP–Hib–hepatitis B and PCV) in a population with very low baseline vaccination coverage was challenging, given that there were only three vaccination rounds and attendance was around 70%. Although our campaign covered a large area, some inequity in accessing vaccinations might have remained and there could have been some low-coverage pockets where people were still vulnerable to vaccine-preventable diseases. Our strategy was to optimize performance by adapting the campaign design to take into account experience gained during each vaccination round. We added vaccination sites, improved communication and collaboration with the community, and provided paracetamol for use only to treat fever or pain in the days following vaccination. To account for situations in which families had access to both the EPI and our preventive vaccination campaign, we adapted our health promotion messages to communities. In addition, our campaign was selective: we took into account vaccinations administered by the EPI that were recorded on cards. We also encouraged families to continue visiting their closest EPI centre regularly for further vaccinations as the campaign was only temporary.

Collaboration between Médecins Sans Frontières and the health ministry during the planning, implementation and evaluation of the campaign was excellent. One challenge was a lack of personnel with sufficient experience of the EPI. Another was a misunderstanding about the vaccines Médecins Sans Frontières needed from the health ministry, which led to the third round of PCV immunization being slightly adapted. Finally, there was the challenge of not depleting the EPI’s personnel by employing health workers seeking additional income, in light of the poor remuneration offered by the health ministry.

As resources are never unlimited, devising a vaccination strategy for remote populations with poor access to the EPI and to medical care presented a dilemma: should overall vaccination coverage be optimized by concentrating efforts in the most populated areas and vaccinating the maximum number of people or should vaccination activities be distributed equitably across the geographical area? Targeting hard-to-reach populations requires more resources per person, but is crucial because these populations have limited access to medical care for disease and because pockets of low vaccine coverage are potential sources of epidemics. Médecins Sans Frontières chose an equitable approach for the campaign and covered remote areas as best as possible, which led to higher costs. Consequently, costs were probably too high for the health ministry to replicate the campaign. It is important, then, that different vaccination strategies are complementary. One option is to increase the number of immunization rounds in some areas according to vaccination coverage data. In addition, linking some supplementary immunization activities to other vaccination programmes could be explored. Deciding on which population groups to target should take into account local epidemiology, vaccination coverage data and the resources available.

In the Central African Republic, a child must attend an EPI site at least four times during the first year of life to complete the EPI schedule (apart from birth vaccines). However, limiting the EPI to children younger than 1 year is unrealistic and fails to achieve satisfactory vaccination coverage.^5^ Extending the EPI to children aged 2 years or more is essential as a catch-up strategy for those with incomplete immunization.

The approach adopted for this campaign and for some others organized by Médecins Sans Frontières in 2014 and 2015 differed from previous campaigns despite similar needs for catch-up vaccinations. Previous campaigns were not preventive; they were primarily reactive campaigns against measles or meningitis outbreaks. The recent development of preventive, multi-antigen campaigns has been made possible by better understanding of the feasibility of such campaigns in humanitarian emergencies and other undervaccinated contexts, by Médecins Sans Frontières’ experience with mass reactive campaigns and by WHO’s recommendations.

In situations where the EPI is weakened, such as in the recent humanitarian crisis in the Central African Republic, a targeted, subnational, preventive, multi-antigen, mass vaccination campaign, as recommended by WHO, can be effective in rapidly increasing immunization coverage and decreasing the accumulation of susceptible children. As a consequence, the risk of an epidemic in the wake of a humanitarian crisis and the burden of disease could be reduced. However, routine EPI activities must be strengthened in parallel and over the long term if increased vaccination coverage is to be sustained. The sustainability of increased coverage could be assessed by regular surveys, which would help guide decisions on more affordable and innovative campaigns.
