# Corrigendum

**DOI:** 10.2471/BLT.18.101118

**Published:** 2018-11-01

In: Peyraud N, Quére M, Duc G, Chèvre C, Wanteu T, Reache S, et al. A post-conflict vaccination campaign, Central African Republic. Bull World Health Organ. 2018 Aug 1;96(8):540–47. http://dx.doi.org/10.2471/BLT.17.204321

on page 544, Fig. 2, should be as follows: 

**Fig. 2:**
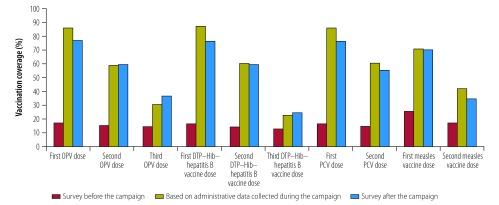
Vaccination coverage of children aged 12 to 23 months before and after preventive mass vaccination campaign, Mambéré-Kadéï prefecture, Central African Republic, 2015–2016

DTP: diphtheria, tetanus and pertussis; Hib: *Haemophilus influenzae* type B; OPV: oral poliomyelitis vaccine; PCV: pneumococcal conjugate vaccine.

Notes: Vaccination history in the surveys was based on the caretaker’s recall and the vaccination card, if available. Administrative data collected during the vaccination campaign did not include data on parallel activities under the Expanded Programme on Immunization.

